# The neural correlates of sustained attention in Dementia with Lewy Bodies patients: a task‐based fMRI study

**DOI:** 10.1002/alz70857_102709

**Published:** 2025-12-25

**Authors:** Micaela Mitolo, Davide Braghittoni, Greta Venturi, Elettra Capogna, Lucia Guidi, Luisa Sambati, Annalisa Bosco, Annalena Venneri, Raffaele Lodi, Caterina Tonon

**Affiliations:** ^1^ University of Parma, Parma, Italy, Italy; ^2^ IRCCS Istituto delle Scienze Neurologiche di Bologna, Bologna, Italy, Italy; ^3^ IRCCS Istituto delle Scienze Neurologiche di Bologna, Bologna, Bologna, Italy; ^4^ IRCCS, Istituto delle Scienze Neurologiche di Bologna, and Dipartimento di Scienze Biomediche e NeuroMotorie (DIBINEM), Università di Bologna, Bologna, Italy; ^5^ University of Bologna, Bologna, Bologna, Italy; ^6^ University of Bologna, Bologna, Italy, Italy

## Abstract

**Background:**

Patients with Dementia with Lewy Bodies (DLB) typically experience attentional deficits [1]. The objective of this study was to evaluate the patterns of functional activity during a sustained attention task in a group of DLB compared with healthy elderly.

**Method:**

A total of 20 DLB patients (8F; mean age (SD) = 74.1 (6.1)) and 20 age‐matched healthy controls (HC) (12F; mean age (SD) = 69 (7.6)) underwent a comprehensive neuropsychological assessment and a brain MRI protocol that included an auditory Psychomotor Vigilance Task (PVT) fMRI paradigm. Differences on neuropsychological tests between the two groups were assessed with ANCOVAs or Mann‐Whitney U tests, according to the data distribution. Results were corrected for multiple comparisons and statistical significance was set at *p* <0.05. The average activation levels for PVT across the entire sample were calculated and utilized as a mask for each participant in the MNI152 space; these activations were then overlaid on the Yeo Buckner atlas [2] to determine the median activations of each brain network.

**Results:**

DLB patients performed worse in neuropsychological tests mainly related to the visuospatial domain (i.e. VOSP subtests, Benton Judgement of line orientation, Rey–Osterrieth complex figure) and had poorer performance on sustained attention (i.e. PVT) in terms of lapses (*p* = 0.01), and longer response time (*p* = 0.01). The pattern of functional activity during the PVT task highlights significantly less activation in the DLB group compared with HC in the right superior lateral occipital cortex, right cuneus and right precuneus (*p* <0.001).

**Conclusion:**

DLB patients performed poorly on tasks within the attentive and visuo‐spatial domains. Slower reaction times in the DLB group were associated with decreased activation in cuneus and precuneus. These data confirm the central role of these brain areas in sustained attention, one of the primary components of attention, as previously shown in healthy young participants [3]. Further studies with larger DLB populations are needed to confirm the functional mechanisms underlying the various components of attentive deficits typically experienced by these patients.

**Acknowledgement**: This study has been supported by the Italian Ministry of Health (#GR‐2019‐12369242) and by #NEXTGENERATIONEU, project MNESYS (PE0000006).

